# The Role of Intestinal Bacteria and Gut–Brain Axis in Hepatic Encephalopathy

**DOI:** 10.3389/fcimb.2020.595759

**Published:** 2021-01-21

**Authors:** Zefeng Chen, Jingsheng Ruan, Dinghua Li, Min Wang, Zhiwei Han, Wenxia Qiu, Guobin Wu

**Affiliations:** Guangxi Medical University Cancer Hospital, Nanning, China

**Keywords:** bile acid, ammonia, neurotransmitter, blood–brain barrier, neuroinflammation, gut microbiota, hepatic encephalopathy

## Abstract

Hepatic encephalopathy (HE) is a neurological disorder that occurs in patients with liver insufficiency. However, its pathogenesis has not been fully elucidated. Pharmacotherapy is the main therapeutic option for HE. It targets the pathogenesis of HE by reducing ammonia levels, improving neurotransmitter signal transduction, and modulating intestinal microbiota. Compared to healthy individuals, the intestinal microbiota of patients with liver disease is significantly different and is associated with the occurrence of HE. Moreover, intestinal microbiota is closely associated with multiple links in the pathogenesis of HE, including the theory of ammonia intoxication, bile acid circulation, GABA-ergic tone hypothesis, and neuroinflammation, which contribute to cognitive and motor disorders in patients. Restoring the homeostasis of intestinal bacteria or providing specific probiotics has significant effects on neurological disorders in HE. Therefore, this review aims at elucidating the potential microbial mechanisms and metabolic effects in the progression of HE through the gut–brain axis and its potential role as a therapeutic target in HE.

## Introduction

Hepatic encephalopathy (HE), typically divided into three types [type A resulting from acute hepatic failure (ALF), type B resulting from portosystemic bypass or shunting, and type C resulting from cirrhosis], is a neurological complication that occurs in individuals with chronic liver diseases (Montagnese et al., 2018). In this condition, the body’s metabolic processes are interrupted by hepatic dysfunction, ammonia, bile acids, and other substances that cross the blood–brain barrier (BBB) with increased permeability, accumulate in the brain, eventually causing neurological disorders. The impaired lymphatic system cannot, however, eliminate harmful substances, which may eventually aid the entire process (Ochoa-Sanchez and Rose, 2018; Hadjihambi et al., 2019). Mild HE (MHE) is a subclinical HE (SHE) that lacks the clinical manifestations associated with HE. Routine mental and neurological tests are normal in SHE. The diagnosis of MHE depends on psychometric and neurophysiological tests. It is very challenging to give accurate clinical diagnosis before overt symptoms set in, thus, leading to a decrease in the quality of life and survival time (Bajaj, 2008; Wijdicks, 2016). Learning ability of HE is not completely reversible and new cognitive decline occurs after treatment in some patients (Riggio et al., 2011; Hopp et al., 2019).

Intestinal microbiota are associated with human digestion and exhibit direct or indirect links to human health (Premkumar and Dhiman, 2018). New treatment modalities aim at regulating the balance of gut microbiota for relieving or curing related diseases. Studies have documented that intestinal bacteria are closely associated with emotional and cognitive–behavioral functions. The gut–liver–brain axis comprehensive treatment concept can be used to manage cognitive–behavioral disorders in HE (Oliphant and Allen-Vercoe, 2019). Intestinal microbiota significantly contribute to the pathogenesis of autism, Alzheimer’s diseases, Parkinson’s disease and other central nervous system (CNS) diseases (Zhu et al., 2020). The gut–brain axis is also involved in the progression of nervous system dysfunction. This review elucidates on the various mechanisms involved, and how intestinal microbiota and its metabolites facilitate the progression of liver diseases to HE through the gut–brain axis, and the potential therapeutic options for HE by regulating intestinal microbial community composition.

## Liver Disease Impacts Intestinal Homeostasis Through The Gut–Liver Axis

Maintenance of intestinal homeostasis is dependent on an intact intestinal mucosal barrier, a healthy intestinal microenvironment, and a delicate balance between nutrition and metabolites. Intestinal dysfunction in patients with HE occurs as liver function deteriorates. Small intestinal bacterial overgrowth and bacterial translocation are the essential features for intestinal homeostatic imbalance in patients with severe liver disease. Gut–liver axis is a pathway for bi-directional communication between the intestine and the liver. Regular operation of the gut–liver axis requires an intestinal mucosa barrier and a healthy liver function. The intestinal barrier is the first barrier against bacteria and their metabolites entering the blood. In some patients with liver disease, the intestinal barrier is destroyed, depending on disease severity (Simbrunner et al., 2019; Albillos et al., 2020; Gerova et al., 2020).

The liver is the body’s largest immune organ. It eliminates toxic substances and bacterial metabolites from the intestines. Enterohepatic circulation of bile acids and urea plays a vital role in the gut–liver axis. The liver and gallbladder secrete primary bile acids into the gut. Various microbial species, including *Lactobacillus, Bifidobacterium* and *Enterococcus*, secrete bile salt hydrolase (BSH) and bile acid dehydratase enzymes that catalyze primary bile acids into secondary bile acids. Most circulating bile acids (about 95%) are taken up by the enterocytes and are recycled into the liver through the portal vein. Bile acids are then discharged into the intestines through the biliary tract after liver metabolism (Long et al., 2017; Mertens et al., 2017).

Elevated blood ammonia (hyperammonemia) levels cause mitochondrial dysfunction, oxidative or nitrative stress and cause apparent damage to the nervous systems such as brain permeability disorders, nerve conduction abnormalities, and the alteration of glucose metabolism in the human brain (Fan et al., 1990; Jayakumar and Norenberg, 2018). The intestinal tract is the primary source of ammonia. Intestinal bacteria decompose protein into ammonia by producing urease. Intestinal ammonia can be absorbed into the bloodstream. After ammonia is transported into the portal vein, it enters the liver and is re-synthesized to urea. This process is called enterohepatic circulation of urea. Urea enterohepatic circulation maintains a low concentration of ammonia in human blood (Wright et al., 2011). When enterohepatic circulation is cut off, the levels of ammonia and bile acids in the blood increase.

If intestinal metabolites in the blood are difficult to be broken down in the liver, or circulate directly through the collateral systems, hence, bypassing the liver, it leads to an increase in the concentration of toxic substances or neurotransmitters in the CNS. Gut bacteria release their components, including lipopolysaccharides (LPSs), peptidoglycan (PGN), bacterial lipoproteins (BLPs), mannans, and bacterial DNA into the blood. Lipopolysaccharide is the main component of gram-negative bacteria that triggers systemic inflammation. Moreover, high amounts of LPS increase BBB permeability and neuroinflammation, causing a large number of bacterial metabolites to get into the brain quickly, thus promoting the occurrence of HE (Hemmi and Akira, 2002; de Jong et al., 2016).

## Intestinal Microbiota Communicate With Central Nervous System Through Gut–Brain Axis

Intestinal bacteria start to colonize the human body after birth. Maternal bacteria colonize the fetus’s intestinal tract during delivery. As the infants mature, their gut microbiota composition improves and resembles that of healthy adults (Perez-Mu Oz et al., 2017). However, some studies have documented that the fetus obtains its gut microbiome or is exposed to microbial products and metabolites from maternal microbiota, which plays a vital role in the fetus’s immune system or metabolism. (Gomez et al., 2016; Younge et al., 2019). Therefore, to maintain a healthy brain function, it is important to understand the relationships between CNS and intestinal microbiome. Germ-free (GF) mice are standard animal models used to study how intestinal microbiota affect the nervous system. Apparent differences in neurological development and neurotransmitter concentration exist between mice with typical microbiota and GF mice, which, however, show that commensal bacteria regulate and control the cognitive and motor functions of the nervous system (Mitsuharu et al., 2013; Principi and Esposito, 2016).

The enteric nervous system (ENS) connects intestinal microbiota with the CNS and is an essential communication pathway for the gut–brain axis. Intestinal microbiota also regulate the development and function of the ENS. Colonizing the intestinal microbiota of conventionally raised mice in GF mice can change the anatomical structure of the ENS and improve the intestinal transport function, which is associated with the intestinal microbial metabolite 5-HT and microorganism activation 5-HT4 receptors in the ENS. Therefore, gut microbiota affects the CNS through the ENS (De Vadder et al., 2018).

Commensal gut microbiota and their metabolic products communicate with the CNS by mediating the activity of the vagus nerve and by regulating endocrine and immune pathways, which in turn exert an impact on cognitive, motor, and nervous system development. Intestinal bacteria affect the structure and function of the CNS. The affected structure and functions involve neurogenesis, myelination, glial cell function, synaptic pruning and BBB permeability of the CNS (Mika and Fleshner, 2015; Bonaz et al., 2018; Heiss and Olofsson, 2019; Nabhani and Eberl, 2020). Beneficial bacteria have been developed as intestinal microecological agents for clinical treatment because they play a significant role in CNS function.

## Changes And Influence of Gut Microbiota In Hepatic Encephalopathy

Intestinal microbiota disorder is characterized by low intestinal microbiota diversity, overgrowth of harmful microbiota, and disruption of beneficial microbiota in HE. Compared to healthy controls, intestinal microbiota of cirrhosis patients has an abundance of 75,245 genes according to quantitative metagenomics (Qin et al., 2014). *The genus Bacteroidetes decreases with a decrease in liver function* (Haraguchi et al., 2019). Some intestinal microbiota have been correlated with the pathological mechanisms, processes and outcomes of HE (Bajaj, 2014; Iebba et al., 2018; Sung et al., 2019). For instance, the translocation of *Stenotrophomonas pavanii* and *Methylobacterium extorquens* into the peripheral blood system enhances the risk of HE (Iebba et al., 2018).

**Figure 1:**
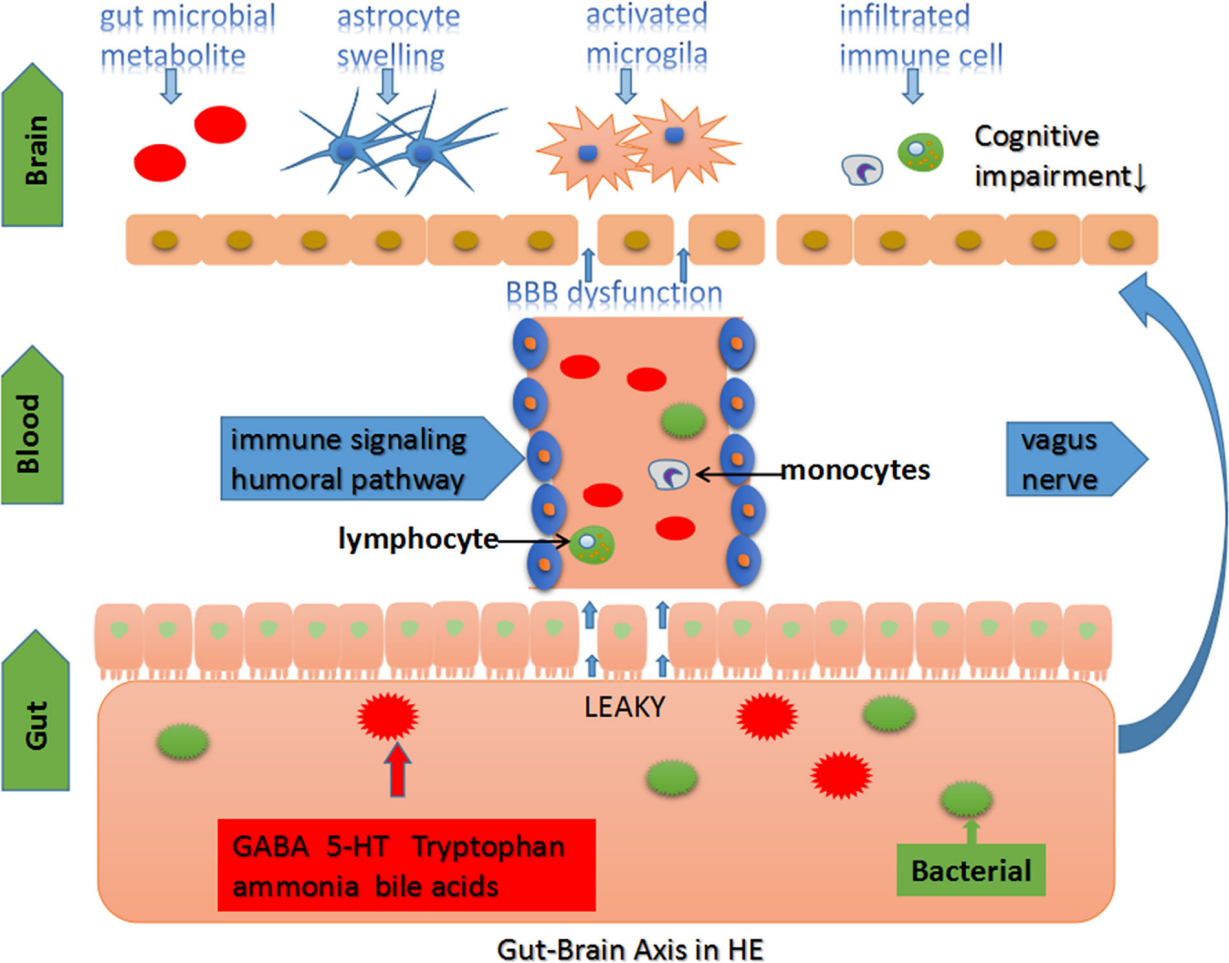
The gut-brain axis in HE. The homeostasis of intestinal microbiota is affected in severe hepatic disease and portal shunt disease. Gut-origin substances are delivered to the brain through the immune, humoral and vagus nerve pathway (D’Mello et al., 2009; Cawthon and de La Serre, 2018). Chronic intestinal inflammation and “leaky gut” promote gut microbiota metabolite and bacterial translocation into the circulatory system leading to systemic inflammation and body metabolic disorders (Seo and Shah, 2012). The brain microenvironment loses stability, followed by BBB dysfunction. Moreover, multiple factors disturb the CNS function, including changes in brain structure, neurotransmitters, and other substance concentrations, leading to cognitive impairments in HE (Jones, 2003; Banks, 2006; Dhanda and Sandhir, 2015; Jayakumar and Norenberg, 2018; DeMorrow, 2019).

Cognitive and motor disorders originate from different structures of an impaired CNS. Psychometric HE score and diffusion kurtosis imaging (DKI) have been used to evaluate cognition and brain microstructure changes of patients with liver cirrhosis, respectively. Compared to healthy individuals, DKI parameters of gray matter and white matter have been found to be significantly decreased in cirrhosis. Psychometric HE score was found to be low and positively correlated with DKI parameters in cirrhosis, indicating that decreased brain microstructural complexity and cognitive impairment in patients with liver cirrhosis may have a potential correlation. (Chen et al., 2017). Thus, the link between microbiota changes and structural brain lesions enhances the understanding of HE. Ahluwalia et al. used magnetic resonance spectroscopy (MRS) and diffusion tensor imaging (DTI) to determine the association between the changes seen in the CNS and microbiota in HE. *Enterobacteriaceae* and *Autochthonoustaxa* were found to be positively and negatively correlated with astrocyte swelling, respectively. Based on the analysis of DTI images, *Porphyromonadaceae* is associated with neuronal damage (Ahluwalia et al., 2016). Moreover, *stool Alcaligenaceae* has been correlated with poor cognition in OHE (Bajaj, 2014). Identifying specific gut microbiota provides new strategies for clinical diagnosis, treatment, and eventually weighing the prognosis of HE. A summary of the above studies is presented in **Table 1**. The table shows the progression, outcomes, and specific microbiome in HE. And microbiota-associated mech-anisms involved in the pathogenesis of HE are showed in **Figure 1**.

**Table 1:** The connection between gut microbiome and HE (positive relation ↑ and negative relation ↓).

Author	HE	Specimen	Method	Bacterial species
(Iebba et al., 2018)	Risk	Fecal	16S sequencing and NMR metabolism	*Bacteroides coprocola*↑ *Bifdobacterium longum*↑ *Bacteroides faecis* ↓ *Bacteroides coprophilus* ↓
		Blood		*Stenotrophomonas pavanii*↑ *Methylobacterium extorquens*↑ *Clostridium indolis*↓
(Sung et al., 2019)	Mortality	Fecal	16S sequencing	*Lactobacillus*↑ *Bacteroides* ↓ *Clostridium incertae sedis*↓ *Clostridium XI* ↓
	Recurrence	Fecal	16S sequencing	*Veilonella*↑ *Phascolarctobacterium* ↓ *Fusobacterium* ↓
(Zhang et al., 2013) (Ahluwalia et al., 2016)	Astrocyte swelling	Fecal	16s sequencing, MTPS	*Autochthonous taxa*↓ *Enterobacteriaceaeand*↑ *S. salivarius*↑
(Ahluwalia et al., 2016)	Neuronal damage	Fecal	MTPS	*Prevotellaceae*↑ *Veillonellaceae*↑ *Porphyromonadaceae*↑/↓

## Blood–Brain Barrier Permeability

Brain edema is a common characteristic in HE that promotes neurological deterioration (Cudalbu and Taylor-Robinson, 2019). Permeability of the BBB increases in patients and animal models of HE (Dhanda and Sandhir, 2018; Weiss et al., 2019). The BBB, which is a crucial regulatory interface in the gut–brain axis, modulates the transportation of immune cells, inflammatory molecules, and intestinal bacterial metabolites, thereby, stabilizing the CNS microenvironment (Banks, 2006). Occludin and claudin-5 are key tight junction proteins that play an important role in regulating BBB permeability. Compared to mice with healthy gut microbiota, BBB permeability was found to be increased in GF mice, which relates to the expression of occludin, and claudin-5. Transplantation of healthy gut microbiota from pathogen-free mice was shown to ameliorate the changes in GF mice (Braniste et al., 2014).

BBB damage in HE patients is associated with the swelling of astrocytes, endothelial cell damage, and the opening of tight junctions. Ammonia and inflammation are responsible for BBB dysfunction in HE (Erickson et al., 2012; Marta and Jan, 2012). Hyperammonemia triggers brain edema by disrupting the glutamate or glutamine cycle in astrocyte (Norenberg and Martinez-Hernandez, 1979). Key connexins form a gap junction between astrocytes. In rats with bile duct ligation (BDL) or hyperammonemia, elevated blood ammonia levels are associated with gap junction dysfunction, which is significantly improved after ammonia-lowering treatment. However, the treatment effect is not mediated by increasing the expression of key connexins (Hadjihambi et al., 2017). Moreover, ammonium chloride was shown to down-regulate claudin-12 gene expression in a brain capillary endothelial cell culture model (Bélanger et al., 2007). Specific membrane transporters form the structural basis for BBB functions and the transportation of specific substances in and out of the brain. P-glycoprotein and Mrp2 are the ATP-binding cassette (ABC) transporters expressed in the brain endothelial cells (ECs) (Ebinger and Uhr, 2006). Expression and function of the ABC transporter affect drug distribution in the brain and prevents the accumulation of endotoxins in the nervous system. Moreover, hyperammonemia increases the expression of P-glycoprotein and Mrp2 by activating the NF-kB pathway in the BBB (Zhang et al., 2014). In ALF, the expression and function of ABC transporters in the BBB are also altered (Fan and Liu, 2018).

A high concentration of LPS results in robust inflammatory responses. Lipopolysaccharides bind brain endothelial cell membrane receptors, including TLR-2, TLR-4, and CD14, causing the release of cytokines and inflammatory mediators. However, a lower concentration of LPS enhances innate immune functions (Singh and Jiang, 2004; Dauphinee and Karsan, 2006). After injection of LPS, ALF mice were found to further aggravate hepatic injury and develop symptoms of liver coma. Moreover, BBB is permeable to immunoglobulin G (IgG), which may be modulated by the up-regulation of MMP9 (Chastre et al., 2014). MMPs are proteases that degrade extracellular matrix (ECM), which can easily lead to an increase in vascular permeability. In BDL rats, MMP9 levels were found to be enhanced in the cortex, hippocampus, and striatum (Dhanda and Sandhir, 2018). Moreover, in LPS-induced systemic inflammatory responses, MMP9 activity was shown to be regulated by Cyclooxygenases-1 and -2 (COX1/COX2), which are critical regulators of innate immune responses (Aid et al., 2010). Banks and colleagues, using *in vitro* BBB models and animal inflammatory models, postulated that LPS-induced disruption of the BBB may be dependent on COX (Banks et al., 2015).

Gut microbial metabolites affect the physiological state of the BBB by producing SCFAs. GF mice exhibited decreased BBB permeability after Clostridium tyrobutyricum transplantation that mainly produces butyrate and after oral gavage of sodium butyrate (Braniste et al., 2014). In addition, propionate can protect the BBB by binding the receptor FFAR3 expressed in the human brain endothelium against oxidative stress (Hoyles et al., 2018).

## Neuroinflammation and Immune Regulation

Neuroinflammation regulates mood and behavior in patients by regulating the basal ganglia, cortical reward, and motor circuits. Anxiety-related areas of the brain are also affected (Felger, 2018; Cabrera-Pastor et al., 2019a). Neuroinflammation of the hippocampus and cerebellum is a key pathological feature of HE that leads to cognitive impairment. Hippocampal volume is decreased in cirrhosis patients. Neuroinflammation affects the expression of hippocampal glutamate receptors and GABAergic tone in the cerebellum, inducing spatial memory or movement disorder (Hassan et al., 2019; Lin et al., 2019).

Peripheral inflammation and chronic hyperammonemia collectively promote neuroinflammation in liver disease. Liver and intestinal function disorders promote the release of peripheral inflammatory factors, which can pass the BBB and directly affect brain functions (Banks, 2005; Rodrigo et al., 2010; Luo et al., 2015). Microglia are the primary immune cells in CNS, and excessive activation of microglia is the primary source of inflammatory factors that cause neuronal damages (Jaeger et al., 2019). The mechanisms of microglial activation include: i. brain infiltration of peripheral immune cells (D’Mello et al., 2009); and ii. activation of blood cytokine receptors in endothelial cells (Dantzer et al., 2008). Chronic hyperammonemia can also cause neuroinflammatory reactions (Rodrigo et al., 2010; Hernández-Rabaza et al., 2016; Balzano et al., 2020) Injecting extracellular vesicles of hyperammonemic rats into the control group causes neuroinflammatory reactions and dyskinesia (Izquierdo-Altarejos et al., 2020) The use of anti-TNFa therapy, which does not pass the BBB, prevents the neuroinflammatory responses induced by hyperammonemia (Balzano et al., 2020). Therefore, the pro-inflammatory effect of hyperammonemia may be mediated by peripheral inflammation.

Intestinal microorganisms are essential factors that cause systemic inflammation and immune activation of liver diseases. Apart from activating microglia in the brain, intestinal bacteria regulate microglia maturation and homeostasis, which corresponds to microglial defects in mice lacking short-chain fatty acids (SCFAs) receptor FFAR2 (Erny et al., 2015). Lipopolysaccharide is a commonly used *in vitro* inflammatory model of glial cells and animal neuroinflammatory modeling agent. Lipopolysaccharides administration transiently elevates blood levels of interleukin6 (IL6) and tumor necrosis factor-alpha (TNFa) (Labrenz et al., 2019). Pro-inflammatory factors combine with the receptors expressed in Cerebral Endothelial Cells (CECs) to produce a secondary messenger, which induces oxidative stress and neuroinflammation (Azhari and Swain, 2018). Compared to mice with healthy microbiota, there is a significant expression of inflammatory factors in the cortex of GF mice. Moreover, GF mice showed robust neuroinflammation and glial cell activation after receiving intestinal microbiota from cirrhotic mice when compared to mice receiving healthy microbiota. These changes were, however, not caused by liver diseases. These experimental results suggest that an imbalance of intestinal bacterial microbiota drives the development of neuroinflammation in cirrhosis mice and may contribute to the occurrence of HE (Liu et al., 2020).

Microbiota stimulates the vagus nerve to affect brain function in a situation where the intestinal barrier is injured by inflammation. Vagal afferent terminals that are located below the intestinal barrier directly receive the signal produced by microbiota to influence host behavior (Cawthon and de La Serre, 2018). Beneficial microorganisms and probiotic species produce bioactive compounds that regulate host mucosal immune or inflammatory responses. The process is, however, advantageous in improving the inflammation signals received from the peripheral system to the CNS. *Lactobacillus* inhibits TNF production by converting the L-histidine in food into histamine, which improves anti-inflammatory or immunoregulatory functions through the H2 receptor (Hemarajata et al., 2013). Furthermore, inhibition of TNFa formation may also protect against acute ammonia intoxication (Pozdeev et al., 2017).

Gut microbes are involved in immune regulation in HE patients (Martín et al., 2014). Probiotic supplementation plays a beneficial role in the immune function of HE individuals by increasing serum neopterin levels and producing reactive oxygen species (Horvath et al., 2016). Single microbial strains play specific modulatory roles in the body’s immune system (Surana and Kasper, 2014; Geva-Zatorsky et al., 2017). The BBB prevents immune cells from freely entering the nervous system, where immune cells are more likely to enter the nervous system, as seen in the brain of dead cirrhosis patients after autopsy. In an animal model of liver inflammation, microglia activated by TNFa signals were shown to produce MCP1 and CCL2, and recruited monocytes expressing CCR2 into the brain, resulting in a significant infiltration of activated monocytes into the brain (D’Mello et al., 2009). However, there are specific immune system changes observed with MHE, such as increased activation of B lymphocytes and all subtypes of CD4+ T lymphocytes (Mangas-Losada et al., 2017). These changes contribute to neuroinflammation and nervous system disorders.

Suppression/regulation of neuroinflammation is crucial for restoring memory and motor ability in patients with liver cirrhosis or HE. Patients with chronic liver disease, such as steatohepatitis, may have neuropsychological symptoms and cognitive impairment before reaching liver cirrhosis (Felipo et al., 2012; Grover et al., 2012; Mondal et al., 2020). Thus, neuroinflammation in patients with chronic liver disease may have occurred in the early stages of the disease. Balzano et al. analyzed brain tissue samples from patients with different degrees of steatohepatitis and cirrhosis. As disease severity progressed, microglia and astrocytes in the brain were gradually activated and mild steatohepatitis was found to be a pathological feature of neuroinflammation (Balzano et al., 2018). Prompt detection of symptoms and timely treatment may reduce HE cases as well as hospitalization rates.

## Intestinal Bacteria Metabolites in The Gut–Brain Axis

### Ammonia

Hyperammonemia patients with or without cirrhosis have a motor and cognitive dysfunction, suggesting that ammonia affects the brain function through underlying mechanisms (Balzano et al., 2020). Ammonia-induced central nervous system toxicity is the main mechanism of HE. Excessive production of ammonia by gut bacteria such as *S. salivarius* contributes to increased ammonia levels in the blood and astrocyte edema (Zhang et al., 2013).

The primary therapeutic approaches of hyperammonemia include reducing ammonia production and promoting ammonia metabolism (Rose, 2012). Some studies have reported that hyperammonemia can be reduced by modifying intestinal microbiota. *Bacillus Lactis* consumes intestinal ammonia and increases overall survival in chronic and ALF mice (Nicaise et al., 2008). Fecal microbiota transplantation (FMT) was shown to attenuate hyperammonemia in HE animal models, which is an accessible and useful treatment option for patients (Kang et al., 2015). Shen et al. modified intestinal microbes to reduce urease activity, and transplanted them into the intestines of mice with liver injury. There was a significant reduction in mice morbidity and mortality (Shen et al., 2015). Moreover, Kurtz et al. modified the oral probiotic *Escherichia coli nissle 1917* in order to create a strain (*SYNB1020*) that produces l-arginine and consumes NH3 in the *vitro* system. *SYNB1020* was shown to decrease systemic hyperammonemia in a mouse model of thioacetamide (TAA)-induced liver injury. Phase I clinical trial showed a significant clinical effect, indicating the further clinical application of *SYNB1020* for hyperammonemia-related diseases (Kurtz et al., 2019).

Ammonia induces peripheral inflammation leading to cognitive disorders. Decreasing blood ammonia levels is beneficial for recovering cognitive impairment (Balzano et al., 2020). Karababa et al. reported that ammonia attenuates inflammatory responses in an astrocyte-dependent manner in co-cultured astrocytes and microglia treated with LPS. Neurosteroids secreted from astrocytes may contribute to the anti-inflammatory effects of ammonia, which may be one of the potential mechanisms for the absence of microglia reactivity in cerebral cortex of patients with liver cirrhosis and HE (Karababa et al., 2017).

### Bile Acids

Bile acids promote lipid digestion as well as absorption and modulate cellular metabolic activities by binding nuclear receptor, including Farnesoid X Receptor (FXR), Pregnane X Receptor (PXR), Vitamin D Receptor (VDR), and the Glucocorticoid Recptor (GR) (Vítek and Haluzík, 2016). Serum bile acids are elevated during cirrhosis. In an HE animal model, activated apical sodium-dependent BA transporter (ASBT) was shown to promote intestinal bile acid reabsorption, which contributed to increased serum bile acid levels (Xie et al., 2018). However, the homeostasis of the bile acid pool has an intricate connection with intestinal bacteria. Fecal bile acid profile is modulated by gut microbiota in cirrhosis. Chenodeoxycholic (CDCA) and *Enterobacteriaceae* show a strong positive correlation. Meanwhile, *Ruminococcaceae* and Deoxycholic acid (DCA) had a positive correlation. After treatment with rifaximin, *Veillonellaceae*, the ratio of primary and secondary BA levels decreased in six early cirrhotics (Kakiyama et al., 2013).

Bile acids directly or indirectly affect BBB permeability. In BDL rat or rat brain microvessel endothelial cell treated with bile acids, the BBB tight junction was damaged by the activation of Rac1 and the downstream phosphorylation of the tight junction protein occludin (Quinn et al., 2014). Sphingosine-1-Phosphate Receptor 2 Signaling regulated by brain bile acids promotes neuroinflammatory responses in HE, leading to microglial activation and elevated CCL2 expression, thus indirectly and ultimately affecting BBB permeability (McMillin et al., 2017). As BBB permeability increases, unconjugated bile acids may passively diffuse into the brain. Serum bile acid levels have no apparent distinction in cirrhosis with or without HE. However, bile acid levels were found to be increased in the cerebrospinal fluid, while toxic bile acids accumulated in the brains of the BDL mouse models. Therefore, nervous system disorders are associated with the toxic effects of bile acids in HE (Weiss et al., 2016; DeMorrow, 2019).

FXR, activated by bile acids and mainly expressed in the neurons, causes HE-related CNS disturbances. Regulation of bile acids may be a potential strategy for the treatment of HE. FXR knockout mice had a high level of hepatitis development, causing a lower concentration of butyrate in the colon. Butyrate supplements can reverse dysfunctional bile acid synthesis and hepatitis (McMillin et al., 2016; Xie et al., 2018). Bile acids act on the nervous system through nuclear receptors, and can also activate the TGR5 membrane receptor to alleviate neuroinflammation in AOM-induced type A HE mice. The TGR5 receptor is generally expressed in human and rodent tissues and is also up-regulated in multiple animal models with liver injury. TGR5 receptors play an active role in regulating liver inflammation, cholestasis, and fibrosis. The TGR5 receptor was found to be up-regulated in the cortex, thus, improving neurological decline in HE mice after activation by TGR5 (Duboc et al., 2014; Keitel et al., 2019). These findings imply the dual role bile acids play in the progression of HE.

### Short-Chain Fatty Acids

Short chain fatty acids produced by intestinal microorganisms, including butyrate, propionate and acetate, protect the integrity of the intestines and reduce intestinal inflammation. Butyrate, as the main component of SCFAs modulates protein tight junctions to enhance gut barrier function. Its abnormal levels are associated with liver disease severity (Brahe et al., 2013; Stilling et al., 2016; Jin et al., 2019).

SCFAs can cross the BBB; therefore, they play a regulatory role in the gut–brain axis (Joseph et al., 2017). In healthy individuals, *Ruminococcaceae* and *Faecalicatena fissicatena* are positively correlated with SCFAs, which are both, however, decreased in cirrhosis patients. SCFAs provide energy for colonic epithelial cell metabolism. However, the ability to convert carbohydrates into SCFAs is diminished in cirrhosis patients (Brahe et al., 2013; Jin et al., 2019). There is a further reduction of SCFAs in cirrhosis with HE. Butyrate has a negative correlation with inflammatory markers and serum endotoxin (Juanola et al., 2019). SCFAs bind the G-protein-coupled receptor 43 (GPR43) to promote the regression of inflammation (Maslowski et al., 2009). Furthermore, SCFAs downregulate system inflammation and regulate neutrophils, macrophages, and other immune cells (Millard et al., 2002; Vinolo et al., 2011). They also have a strong anti-inflammatory effect on microglial and astrocyte models *in vitro;* therefore, SCFAs may have some potential for regulating neuroinflammatory processes (Huuskonen et al., 2004; Stilling et al., 2016).

### Neurotransmitter

#### Gamma-Aminobutyric Acid

GABA is an important bioactive compound and a crucial inhibitory neurotransmitter in the nervous system. It is mainly produced in the gut by *Bifidobacterium* and *Lactobacillus*, although the GABAergic neurons also produce a small amount of GABA (Yunes et al., 2016; Strandwitz et al., 2019). *Lactobacillus* can regulate GABA concentrations and the expression of GABA receptors in the CNS through the gut–brain axis (Barros-Santos et al., 2020; Chen et al., 2020). In feces, the increased abundance of *Bifidobacterium longum* enhances the risk of HE (Iebba et al., 2018). Elevated GABA levels are associated with physiological and psychological processes in HE (Jones, 2003). When liver failure occurs, serum and brain GABA levels are elevated. GABA can exert pre- and post-synaptic inhibition, leading to motor and consciousness disorders (Kaupmann et al., 1997; Kullmann et al., 2005). Antagonists of the GABA receptor complex can improve the clinical symptoms of HE animals and electroencephalographic abnormalities (Bosman et al., 1991).

Gut ammonia is considered as an essential factor in elevated GABAergic tone. A study by Cauli et al. found out that hyperammonemia selectively increased the GABAergic tone of the cerebellum, ventral thalamus, and the ventromedial thalamus in hyperammonemic rats (Cauli et al., 2009a). The underlying mechanism by which ammonia increases GABA concentration is associated with GABA transaminase activity or neuronal tricarboxylic acid cycle (Palomero-Gallagher and Zilles, 2013). Moreover, Fried et al. reported that ammonia enhances the release of GABA from enteric glia, subsequently altering intestinal neurotransmission, resulting in intestinal motility disorders and an increase in gut ammonia levels (Fried et al., 2017). Studies have also established that changes in GABAA receptor density are up-regulated in hyperammonemia models. In hyperammonemia, elevated GABA concentration and GABAA receptor density correlate to promote CNS disorders, although the expression of the GABAA receptor subunit is not consistent. For instance, GABAA receptor subunit a1 was found to be increased while the alpha-5 subunit was reduced in the hyperammonemia rat model (Palomero-Gallagher and Zilles, 2013; Hernández-Rabaza et al., 2016).

The benzodiazepine receptor (BZR) is part of the GABAA receptor complex; hence exogenous and endogenous benzodiazepine substances bind to GABAA receptor, causing an allosteric regulation of the receptor, thus increasing GABA transport. Ammonia has also been shown to associate with BZR ligands, causing CNS function disorders. Therefore, decreasing ammonia concentration can improve enhanced GABA-ergic (Helewski et al., 2003; Jones, 2003). Clinically, the benzodiazepine receptor is used as the target for improving GABA-ergic. Flumazenil is a benzodiazepine receptor blocker for the treatment of HE. Increased benzodiazepine receptor ligand significantly enhances GABA inhibition in HE brain (Ahboucha and Butterworth, 2005). In HE rat models, BZR levels were not altered in normal rat plasma upon antibiotic intervention. It, however, increased BZR precursors, which may either arise from gut bacteria, increased BZR synthesis in the brain, or enhanced GABA-ergic neurotransmission to promote HE (Yurdaydin et al., 1995).

#### Glutamate

Glutamate is an excitatory neurotransmitter that regulates nervous system development through NMDA and AMPA receptors (Martinez-Lozada and Ortega, 2015). When ammonia levels increase in the brain, glutamate binds ammonia, forming glutamine under the catalytic activity of glutamine synthetase. Accumulation of glutamine and ammonia is associated with brain edema. Studies have found that the extracellular concentration of glutamate increases due to abnormal uptake, transport and release of glutamate. Learning and memory impairment is associated with the abnormal glutamate-NO-cGMP metabolic pathway in the brain (Dabrowska et al., 2018; Cabrera-Pastor et al., 2019b). Even though different concentrations and duration of ammonia have different effects on the expression of glutamate receptors, acute hyperammonemia associated mortalities are mediated by activated NMDAR (Monfort et al., 2002; Kosenkov et al., 2018). NMDAR antagonists were shown to effectively reduce hyperammonemia or ALF induced mortalities in rats (Cauli et al., 2009b; Cauli et al., 2014). Glutamate produced by diet or bacteria cannot be used by the CNS because of BBB. Studies have shown that glutamate and glutamate receptors affect the gut-brain axis in other diseases, such as inflammatory bowel disease (IBD) (Baj et al., 2019). In addition, probiotics and prebiotics can adjust the NMDA/AMPA ratio to affect cognitive functions in middle-aged rats (Romo-Araiza et al., 2018). However, it has not been established whether specific intestinal bacteria alterations affect glutamatergic transmission in HE patients.

### 5-Hydroxytryptamine

Gut tract is the leading site for 5-HT synthesis. High colonic and blood 5-HT levels are associated with specific gut microbiota metabolites (Roshchina, 2010), although the mechanism of 5-HT synthesis that is regulated by microbiota has not been established. Indigenous spore-forming bacteria (Sp) from mouse and human microbiota act on colonic enterochromaffin cells (ECs) to produce 5-HT (Yano et al., 2015). Moreover, probiotics can stimulate the gut–brain axis and increase 5-HT and serotonin transporter (5-HTT) expression, which may promote brain development and function (Ranuh et al., 2019).

Dysfunction of 5-HT receptor and excess serotonergic brain activity is involved in HE development (Apelqvist et al., 1998; Dhanda and Sandhir, 2015; Khiat et al., 2019). In hyperammonemia mice, the 5-HT2B receptor was found to be up-regulated in the brain and had no response to 5-HT. Moreover, the dysfunction of the 5-HT2B receptor was also observed in ammonia treated astrocytes *in vitro* (Yue et al., 2019). 5-HT(1A) is also involved in cognitive–behavioral disorders in HE, while its activation can, reverse nervous system dysfunctions (Magen et al., 2010). However, there was no difference in 5-HT of GF mice before and after FMT (Maslowski et al., 2009). Decreasing peripheral 5-HT absorption can help improve CNS disease. Oral Selective serotonin reuptake inhibitors (SSRIs) improve depression and decreases mortality rates in patients with chronic liver disease. This therapeutic effect is achieved by activating the vagus nerve dependent gut-brain signaling (Mullish et al., 2014; Neufeld et al., 2019).

5-HT concentration depends on the level of tryptophan in the brain (Maslowski et al., 2009). Free tryptophan (TRP), which is the precursor of the neurotransmitter 5-HT, increases only in HE, with no changes in hepatitis and cirrhosis. However, tryptophan is an essential amino acid that competes with other amino acids to cross the BBB. Thus, elevated serum free tryptophan levels invariably increases its availability to the brain and to the activity of serotonergic neurons (Herneth et al., 1998; Lozeva-Thomas, 2004; Saleem et al., 2008). Dietary tryptophan restriction improves neuroinflammation by impairing encephalitogenic T cell responses (Sonner et al., 2019). Probiotic treatment of hyperammonemia rats was shown to significantly decrease 5-HT metabolism (Luo et al., 2014).

## Effects of Clinical Treatment on The Intestinal Metabolome in Hepatic Encephalopathy

Treatments for HE target disease causing agents, control infections, reduce absorption of intestinal ammonia, and correct the metabolic dysfunction caused by liver diseases. Several drugs, including antibiotics and laxatives, are used to treat HE. Probiotics and other drugs are also used in clinical practice. Clinical therapeutic drugs may or may not alter the intestinal metabolome to achieve therapeutic effect. We discuss the effects of several commonly used drugs on the intestinal microbiota of HE patients.

### Antibiotics

Rifaximin is a common antibiotic used to treat patients with HE. It improves hyperammonemia, endotoxemia, and cognitive dysfunction (Kaji et al., 2017). Other antibiotics such as neomycin are not recommended because of their side effects (Patidar and Bajaj, 2013). Although rifaximin has bactericidal and bacteriostatic effects, it does not change the abundance of dominant intestinal bacteria in HE patients. In addition, it does not control the abundance of Gram-negative bacteria. It decreases blood endotoxin levels through unknown mechanisms. It is postulated that it can regulate the metabolism of intestinal bacteria or stabilize intestinal barrier functions (Kaji et al., 2017; Montagnese et al., 2018).Other studies have shown that it has immunomodulatory effects as it reduces inflammation by regulating bacteria. Rifaxmin was shown to improve the immune system in 59% of MHE patients (Mangas-Losada et al., 2019).

### Lactulose

Various drugs are used to reduce blood ammonia levels. Lactulose is the most commonly used ammonia-reducing drug. It is an unabsorbable disaccharide that is used as a laxative because it triggers the production of large amounts of ammonia in stool (Montagnese et al., 2018). Lactulose alone or in combination with rifaximin is widely used in the treatment of HE. Clinical studies have shown that lactulose improves patients’ cognitive functions and quality of life (Wang et al., 2019). In rats with CCL4-induced ALF, lactulose improved the plasticity of the nervous system (Yang et al., 2015). It also inhibits intestinal bacterial overgrowth, translocation and intestinal resistance. This decreases systemic inflammatory responses and hyperammonemia in HE rats. In patients with liver cirrhosis, clinical doses of lactulose promote the growth of beneficial bacteria, such as *Bifidobacterium* and *Lactobacillus* (Montagnese et al., 2018).

### Probiotic

Probiotic treatment is a new adjuvant therapy for HE. Clinical studies have shown that probiotics can prevent the occurrence and recurrence of HE in patients with cirrhosis (Lunia et al., 2014; Venigalla et al., 2015). Probiotics comprise various bacteria which directly improve the composition of intestinal microbiota, thereby, conferring therapeutic effects. Probiotics reduce bacterial ammonia production and the absorption of intestinal ammonia and other toxins (Solga, 2003). In patients with compensatory cirrhosis taking multiple probiotic strains for 6 months, their stool was found to be rich in probiotic strains, including *Lactobacillus brevis*, *Lactobacillus salivarius* and *Lactococcus lactis*. In addition, probiotics may boost the production of short-chain acids by increasing the abundance of multiple bacteria, including *Calibacterium prausnitzii*, *Syntrophococcus sucromutans* and *Alistipes shahii* (Horvath et al., 2020).

### Fecal Microbiota Transplant

FMT is an emerging treatment approach that is aimed at rebuilding intestinal microbiota to treat diseases, and is gradually being generalized for the treatment of various intestinal dysfunction diseases, such as inflammatory bowel disease (IBD) (Monfort et al., 2002; Bibbo et al., 2017). A few animal experiments have shown that FMT has obvious protective effects on CCL4-induced ALF rats (Wang et al., 2017). This beneficial effect is not only observed in the improvement of cognitive function, but can also improve the markers of disease activity associated with the gut-liver-brain axis disorder. FMT was shown to significantly reduce neuroinflammatory responses in CCL4-induced cirrhotic mice. It also provided effective protection in HE by restoring normal intestinal permeability and improving liver damage indicators. TOLL-like receptors are important mediators of inflammatory responses. Hepatic TLRs and serum ammonia levels were found to be significantly down-regulated in cirrhosis rats after FMT (Wang et al., 2017; Liu et al., 2020). Although clinical trials of FMT are ongoing, we discussed its effectiveness and safety in clinical treatment based on the published results.

Recurrent HE leads to hospitalization. In an open and randomized clinical trial, it was determined whether the therapeutic effect of FMT enema in cirrhosis patients with recurrent HE after pretreatment with antibiotics is better than standard of care (SOC). Compared to SOC, a reasonable choice of donor FMT can significantly improve cognitive functions in patients and reduce incidences of serious adverse events. In a randomized, single-blind, placebo-controlled phase 1 clinical trial, compared to placebo, oral FMT capsules showed significant safety in cirrhosis patients with recurrent HE (Bajaj et al., 2017; Fuchs and Puri, 2020). Fecal transplantation improves liver functions in a number of liver diseases (Lechner et al., 2020). Restoring liver functions reduces the impact of various factors on the nervous system, prevents or defers neurological disorders in patients with liver diseases, and improves the quality of life for patients. Although clinical trials involving different liver diseases have just begun, FMT is an effective treatment method for liver diseases and their complications.

## Summary

Intestinal microbes have been implicated in shaping the nerves and immune systems or other fundamental process during growth. The occurrence of numerous diseases is accompanied by significant changes in microbial communities. As cirrhosis progresses, the composition of intestinal microbiome is altered. Harmful intestinal bacteria promote the occurrence of complications related to liver cirrhosis, including endotoxemia, infection, organ failure, and death. Gut bacteria regulate numerous metabolic processes and physiological functions by secreting different metabolites. Many intestinal metabolites (such as bile acids) are necessary for the human body and undergo enterohepatic recycling, while intestinal metabolic wastes (such as ammonia) are excreted from the body after hepatic metabolism. When these substances exceed physiological concentrations, they produce clinical manifestations of toxicity. They pass the BBB with increased permeability, destroy the nervous system microenvironment, nerve conduction, and even directly lead to coma and death. Intestinal intervention may be a treatment option for all stages of liver disease as it reduces the exposure of the liver and nervous system to intestinal toxins.

Intestinal microbiota is closely associated with CNS function, including brain structure, gene expression, and substance metabolism. Understanding the function of intestinal microbiota in host behavior will promote the management of mental and psychological diseases. Therapies that balance intestinal microbiota are critical for correcting central nervous activity and function in patients with CNS dysfunction due to abnormal intestinal microbiota composition. Such therapies can be designed to target species associated with disease progression. Probiotics or fecal transplantation can be used to manipulate the intestinal microbiome to improve hyperammonemia and endotoxemia. Proper selection of donor FMT reduces hospitalization rates, improves cognition and malnutrition in patients with cirrhosis. It also improves the prognosis of HE patients. Consequently, the underlying mechanisms through which microbes modulate CNS *via* the gut–brain axis should be studied. Liver function alterations in patients with cirrhosis are difficult to reverse. Maintaining intestinal homeostasis to treat liver disease-related nervous system damage is a new potential treatment method. Reasonable intestinal intervention combined with drug treatment may achieve mutually beneficial effects.

## Author Contributions

All authors contributed to the article and approved the submitted version.

## Conflict of Interest

The authors declare that the research was conducted in the absence of any commercial or financial relationships that could be construed as a potential conflict of interest.
